# The genome sequence of the eyed flat-backed millipede, *Nanogona polydesmoides *(Leach, 1814)

**DOI:** 10.12688/wellcomeopenres.24341.2

**Published:** 2026-10-15

**Authors:** Christian Owen, Olga Sivell, Duncan Sivell, Richard J. Twitchett, Gregory D. Edgecombe, Natural History Museum Genome Acquisition Lab, Darwin Tree of Life Barcoding collective, Wellcome Sanger Institute Tree of Life Management, Samples and Laboratory team, Wellcome Sanger Institute Scientific Operations: Sequencing Operations, Wellcome Sanger Institute Tree of Life Core Informatics team, Tree of Life Core Informatics collective, Darwin Tree of Life Consortium

**Affiliations:** 1 Independent researcher, Aberbargoed, Caerphilly, Wales, UK; 2 Natural History Museum, London, England, UK

**Keywords:** Nanogona polydesmoides, eyed flat-backed millipede, genome sequence, chromosomal, Chordeumatida

## Abstract

We present a genome assembly from a specimen of *Nanogona polydesmoides* (eyed flat-backed millipede; Arthropoda; Diplopoda; Chordeumatida; Craspedosomatidae). The genome sequence has a total length of 406.26 megabases. Most of the assembly (95.49%) is scaffolded into 16 chromosomal pseudomolecules. The mitochondrial genome has also been assembled, with a length of 16.55 kilobases.

## Species taxonomy

Eukaryota; Opisthokonta; Metazoa; Eumetazoa; Bilateria; Protostomia; Ecdysozoa; Panarthropoda; Arthropoda; Mandibulata; Myriapoda; Diplopoda; Chilognatha; Helminthomorpha; Eugnatha; Chordeumatida; Craspedosomatidae; *Nanogona*; *Nanogona polydesmoides* (Leach, 1814) (NCBI: txid2794005).

## Background

*Nanogona polydesmoides* ( Leach, 1813–1815) has an Atlantic distribution, being common and widespread throughout Britain and Ireland, ranging as far north as the Shetlands. Continental records are mostly in France, although it is also known from a few sites in Belgium ( Kime, 2004) and Germany ( Decker & Voigtländer, 2010), coastal Norway and Sweden, and an isolated population named as a subspecies in the Italian Alps ( Lee, 2006).

Occurrence data that follow are from Lee (2006), unless otherwise indicated. It is found in a wide range of habitats, although often associated with deciduous woodland. Other habitats with abundant records (in descending order) are maritime, waste ground (with which it is strongly associated, particularly for urban records), grassland, and cultivation. Inland records greatly exceed coastal. It is the most commonly reported millipede in British caves, and this is likewise a common habitat in southern Europe. Typical microhabitats are under stones, fallen logs and bark ( Blower, 1985). It has a slight preference for calcareous soil, most commonly loam or clay. Adults are collected year-round but the autumn and winter account for most records. It is generally considered to be an annual species, laying eggs from November to January ( Blower, 1985), at developmental stadium IX ( David, 1992). Moulting chambers of silken tent-shaped nests have been described from both summer and winter ( Blower, 1985; Main, 1931).

The ‘wing-like’ paranota of *Nanogona* resemble those of polydesmids, but the presence of ocelli in the former readily allows identification. *Nanogona polydesmoides* is the only British species of the genus, which has five other species in western Europe. It is evenly coloured fawn to dark brown, with 30 body rings and a length of 17–21 mm ( Blower, 1985). The body rings bear a distinctive 3 + 3 setae, one on each half of the dorsum and two on each paranota.

Few whole genome sequences for millipedes have been generated; these consist of *Trigonoiulus corallinus* (GCA_013389805.1 ( Kenny *et al.*, 2015), *Helicorthomorpha holstii* (GCA_013389785.1) ( Qu *et al.*, 2020), and of *Glomeris maerens* (GCA_023279145.1), *Anaulaciulus tonginus* (GCA_023279205.1) and *Niponia nodulosa* (GCA_023159045.1) ( So *et al.*, 2022). The MetaInvert database at Senckenberg Görlitz and the LOEWE Centre for Translational Biodiversity Genomics provide draft genome sequences for many soil invertebrates, among them 23 Diplopoda, including three genera/species of Chordeumatida ( Collins *et al.*, 2023).

The genome of *Nanogona polydesmoides* ( Figure 1) was sequenced as part of the Darwin Tree of Life Project, a collaborative effort to sequence all named eukaryotic species in the Atlantic Archipelago of Britain and Ireland. Here we present a chromosomally complete genome sequence for *Nanogona polydesmoides*, based on one adult specimen from Graig, Aberbargoed, Wales, UK (latitude 51.7, longitude −3.23). The genome is of interest because it is the first for the chordeumatid suborder Craspedosomatidea, the three others available being from the Chordeumatidea.

**Figure 1.**
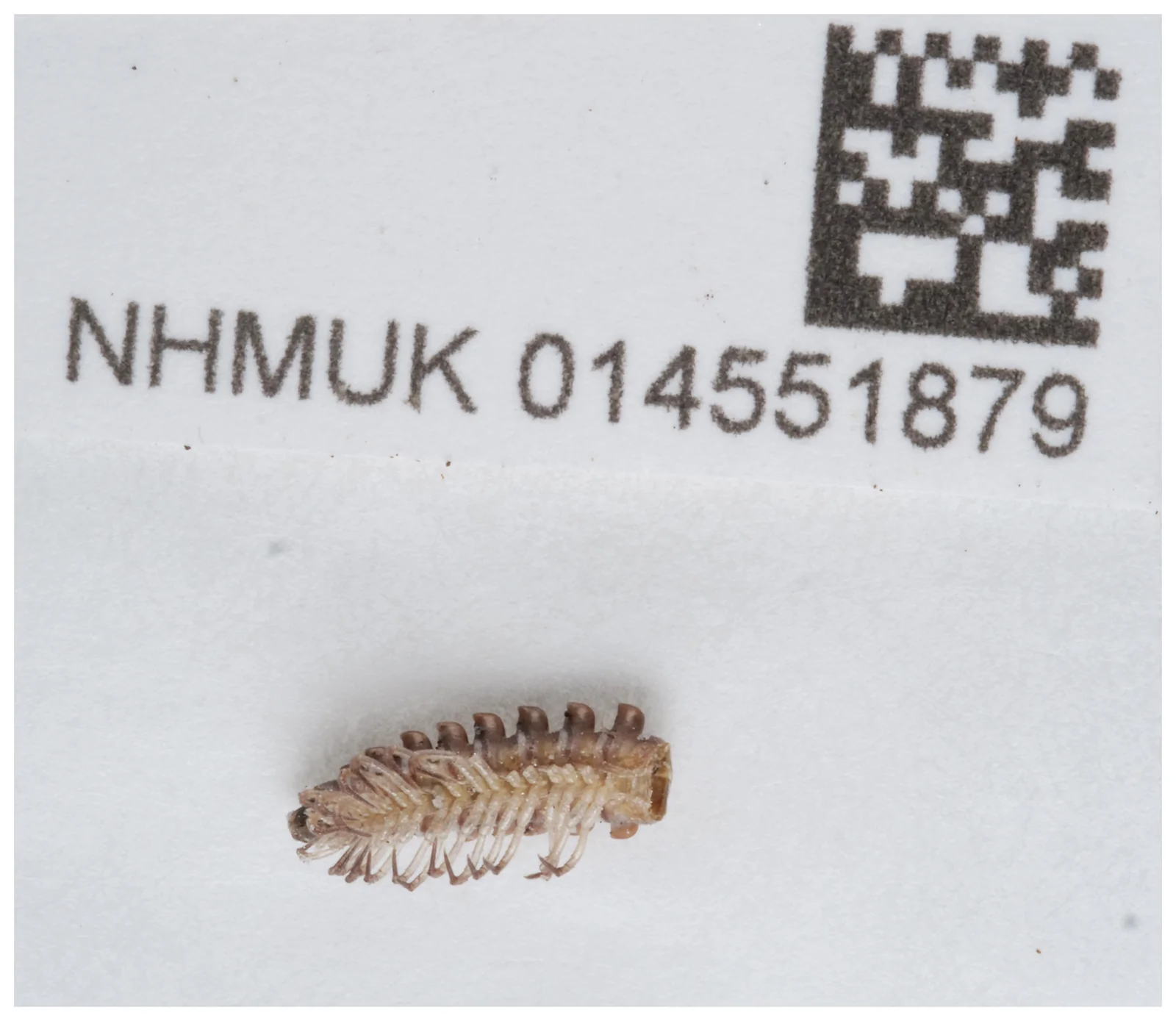
Photograph of the *Nanogona polydesmoides* (qdNanPoly8) specimen used for genome sequencing.

## Genome sequence report

### Sequencing data

The genome of an adult specimen of *Nanogona polydesmoides* ( Figure 1) was sequenced using Pacific Biosciences single-molecule HiFi long reads, generating 32.13 Gb (gigabases) from 4.57 million reads. GenomeScope analysis of the PacBio HiFi data estimated the haploid genome size at 567.03 Mb, with a heterozygosity of 0.88% and repeat content of 37.16%. These values provide an initial assessment of genome complexity and the challenges anticipated during assembly. Based on this estimated genome size, the sequencing data provided approximately 50× coverage of the genome. Chromosome conformation Hi-C sequencing produced 114.19 Gb from 756.19 million reads. Table 1 summarises the specimen and sequencing information.

**Table 1.** Specimen and sequencing data for *Nanogona polydesmoides.*

Project information			
Study title	Nanogona polydesmoides		
**Umbrella BioProject**	PRJEB80243		
**Species**	*Nanogona polydesmoides*		
**BioSpecimen**	SAMEA112222325		
**NCBI taxonomy ID**	2794005		
**Specimen information**			
**Technology**	**ToLID**	**BioSample accession**	**Organism part**
**PacBio long read sequencing**	qdNanPoly8	SAMEA112222384	whole organism
**Hi-C sequencing**	qdNanPoly1	SAMEA7849485	head and anterior body
**RNA sequencing**	qdNanPoly4	SAMEA8534484	Mid-body
**Sequencing information**			
**Platform**	**Run accession**	**Read count**	**Base count (Gb)**
**Hi-C Illumina NovaSeq 6000**	ERR13702777	7.56e+08	114.19
**PacBio Revio**	ERR13698323	4.57e+06	32.13

### Assembly statistics

The primary haplotype was assembled, and contigs corresponding to an alternate haplotype were also deposited in INSDC databases. The assembly was improved by manual curation, which corrected 73 misjoins or missing joins and removed 44 haplotypic duplications. These interventions reduced the total assembly length by 18.06%, decreased the scaffold count by 53.41%, and increased the scaffold N50 by 7.65%. The final assembly has a total length of 406.26 Mb in 320 scaffolds, with 873 gaps, and a scaffold N50 of 25.38 Mb ( Table 2).

**Table 2.** Genome assembly data for *Nanogona polydesmoides.*

Genome assembly		
Assembly name	qdNanPoly8.1	
Assembly accession	GCA_965153395.1	
*Alternate haplotype accession*	*GCA_965153385.1*	
Assembly level for primary assembly	chromosome	
Span (Mb)	406.26	
Number of contigs	1,193	
Number of scaffolds	320	
Longest scaffold (Mb)	32.53	
**Assembly metric**	**Measure**	***Benchmark***
Contig N50 length	0.78 Mb	*≥ 1 Mb*
Scaffold N50 length	25.38 Mb	*= chromosome N50*
Consensus quality (QV)	Primary: 59.3; alternate: 58.5; combined: 58.8	*≥ 40*
*k*-mer completeness	Primary: 73.20%; alternate: 55.74%; combined: 84.91%	*≥ 90%*
BUSCO ^*^	C:96.1%[S:93.6%,D:2.5%], F:2.4%,M:1.6%,n:1,013	*S > 90%; D < 5%*
Percentage of assembly mapped to chromosomes	95.49%	*≥ 90%*
Sex chromosomes	None	*localised homologous pairs*
Organelles	Mitochondrial genome: 16.55 kb	*complete single alleles*

* BUSCO scores based on the arthropoda_odb10 BUSCO set using version 5.5.0. C = complete [S = single copy, D = duplicated], F = fragmented, M = missing, n = number of orthologues in comparison.

The snail plot in Figure 2 provides a summary of the assembly statistics, indicating the distribution of scaffold lengths and other assembly metrics. Figure 3 shows the distribution of scaffolds by GC proportion and coverage. Figure 4 presents a cumulative assembly plot, with separate curves representing different scaffold subsets assigned to various phyla, illustrating the completeness of the assembly.

**Figure 2.**
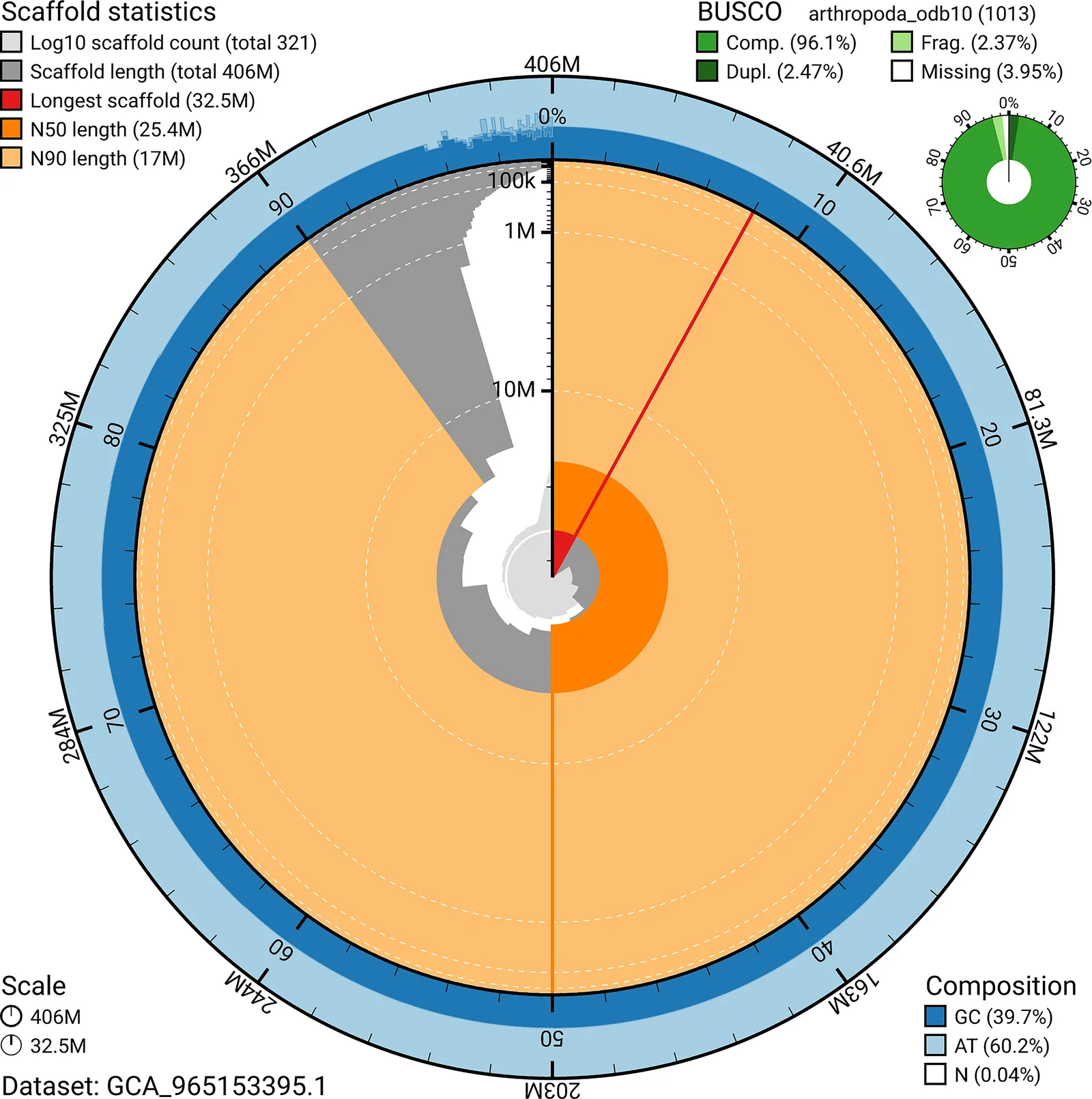
Genome assembly of *Nanogona polydesmoides*, qdNanPoly8.1: metrics.

**Figure 3.**
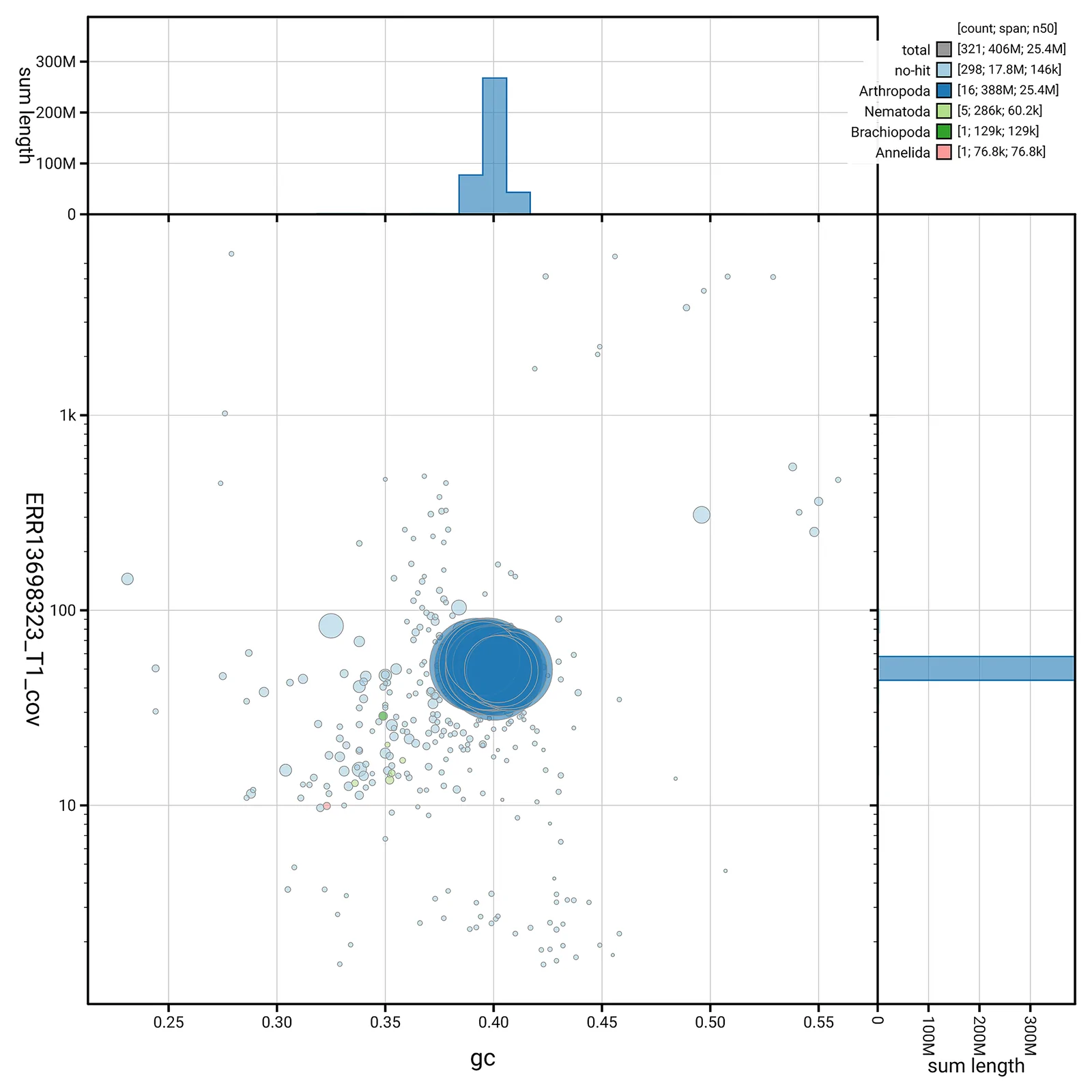
Genome assembly of *Nanogona polydesmoides*, qdNanPoly8.1: BlobToolKit GC-coverage plot.

**Figure 4.**
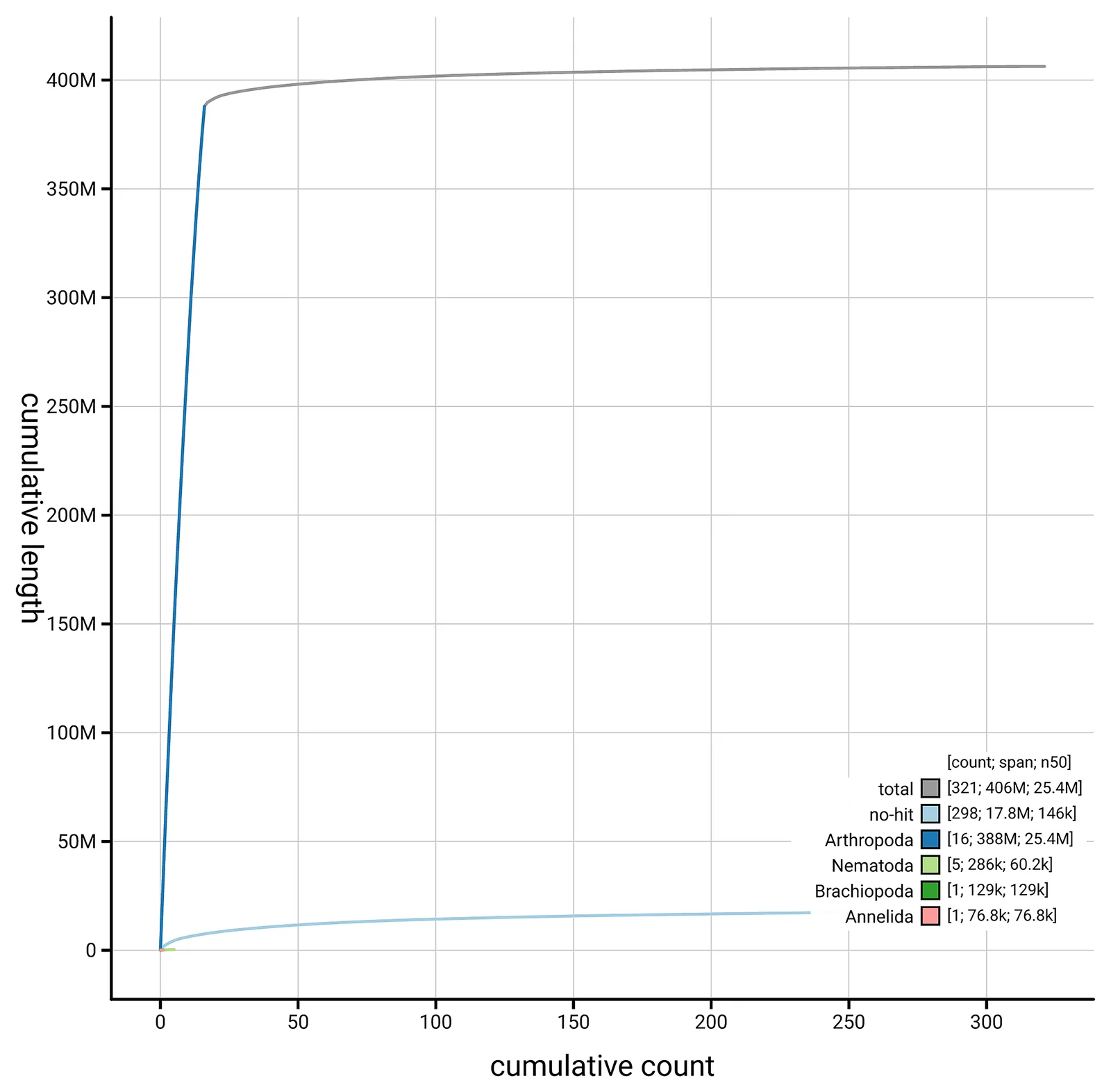
Genome assembly of *Nanogona polydesmoides,* qdNanPoly8.1: BlobToolKit cumulative sequence plot.

The BlobToolKit snail plot provides an overview of assembly metrics and BUSCO gene completeness. The circumference represents the length of the whole genome sequence, and the main plot is divided into 1,000 bins around the circumference. The outermost blue tracks display the distribution of GC, AT, and N percentages across the bins. Scaffolds are arranged clockwise from longest to shortest and are depicted in dark grey. The longest scaffold is indicated by the red arc, and the deeper orange and pale orange arcs represent the N50 and N90 lengths. A light grey spiral at the centre shows the cumulative scaffold count on a logarithmic scale. A summary of complete, fragmented, duplicated, and missing BUSCO genes in the arthropoda_odb10 set is presented at the top right. An interactive version of this figure is available at https://blobtoolkit.genomehubs.org/view/GCA_965153395.1/dataset/GCA_965153395.1/snail.

Blob plot showing sequence coverage (vertical axis) and GC content (horizontal axis). The circles represent scaffolds, with the size proportional to scaffold length and the colour representing phylum membership. The histograms along the axes display the total length of sequences distributed across different levels of coverage and GC content. An interactive version of this figure is available at https://blobtoolkit.genomehubs.org/view/GCA_965153395.1/dataset/GCA_965153395.1/blob.

The grey line shows cumulative length for all scaffolds. Coloured lines show cumulative lengths of scaffolds assigned to each phylum using the buscogenes taxrule. An interactive version of this figure is available at https://blobtoolkit.genomehubs.org/view/GCA_965153395.1/dataset/GCA_965153395.1/cumulative.

Most of the assembly sequence (95.49%) was assigned to 16 chromosomal-level scaffolds. These chromosome-level scaffolds, confirmed by Hi-C data, are named according to size ( Figure 5; Table 3). No sex chromosome could be identified.

**Figure 5.**
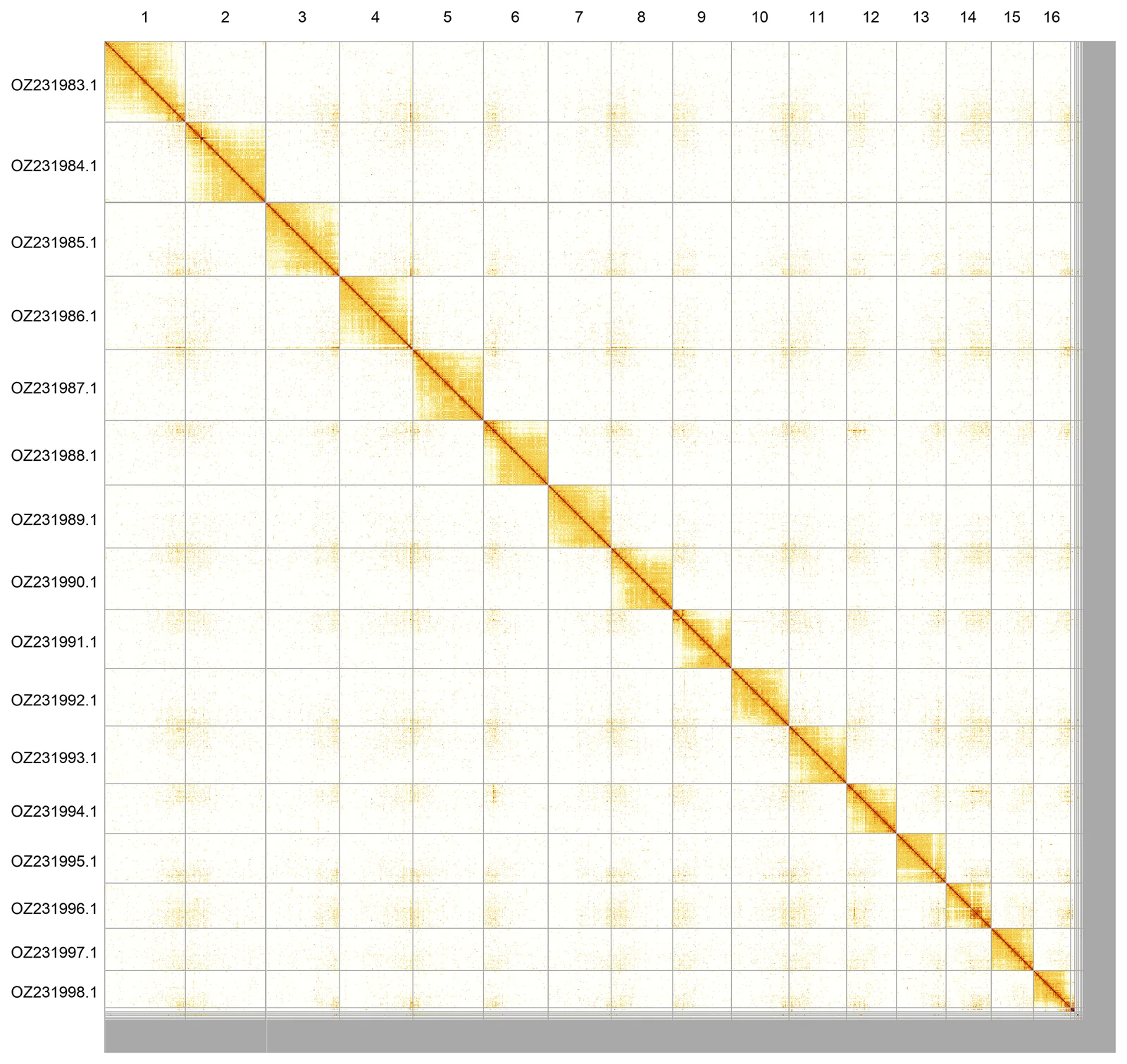
Genome assembly of *Nanogona polydesmoides*, qdNanPoly8.1: Hi-C contact map produced in PretextView.

**Table 3.** Chromosomal pseudomolecules in the genome assembly of *Nanogona polydesmoides*, qdNanPoly8.

INSDC accession	Name	Length (Mb)	GC%
OZ231983.1	1	32.53	40
OZ231984.1	2	32.18	39.5
OZ231985.1	3	29.53	39
OZ231986.1	4	29.47	39.5
OZ231987.1	5	28.36	39.5
OZ231988.1	6	25.98	40
OZ231989.1	7	25.38	40
OZ231990.1	8	24.68	40
OZ231991.1	9	23.58	39.5
OZ231992.1	10	23.16	40.5
OZ231993.1	11	23.09	41
OZ231994.1	12	20.03	40
OZ231995.1	13	19.93	40.5
OZ231996.1	14	18.2	39.5
OZ231997.1	15	17.01	40.5
OZ231998.1	16	14.85	40
OZ231999.1	MT	0.02	28

Chromosomes are shown in order of size from left to right and top to bottom.

The mitochondrial genome was also assembled. This sequence is included as a contig in the multifasta file of the genome submission and as a standalone record.

### Assembly quality metrics

The estimated Quality Value (QV) and *k*-mer completeness metrics, along with BUSCO completeness scores, were calculated for each haplotype and the combined assembly. The QV reflects the base-level accuracy of the assembly, while *k*-mer completeness indicates the proportion of expected *k*-mers identified in the assembly. BUSCO scores provide a measure of completeness based on benchmarking universal single-copy orthologues.

The combined primary and alternate assemblies achieve an estimated QV of 58.8. The *k*-mer recovery for the primary haplotype is 73.20%, and for the alternate haplotype 55.74%; the combined primary and alternate assemblies have a *k*-mer recovery of 84.91%. BUSCO v.5.5.0 analysis using the arthropoda_odb10 reference set ( *n* = 1,013) identified 96.1% of the expected gene set (single = 93.6%, duplicated = 2.5%).

Table 2 provides assembly metric benchmarks adapted from Rhie *et al.* (2021) and the Earth BioGenome Project Report on Assembly Standards September 2024. The primary assembly achieves the EBP reference standard of **5.C.Q59.**

## Methods

### Sample acquisition and DNA barcoding

Metadata collection for samples adhered to the Darwin Tree of Life project standards described by Lawniczak *et al*. (2022). The adult specimen of *Nanogona polydesmoides* used for PacBio DNA sequencing (specimen ID NHMUK014551879, ToLID qdNanPoly8) was collected from Graig, Aberbargoed, Wales, UK (latitude 51.7, longitude −3.23) on 2021-11-27. The specimen was collected and identified by Christian Owen (British Myriapod and Isopod Group) and preserved by dry freezing (−80 °C).

The specimen used for Hi-C sequencing (specimen ID NHMUK014444875, ToLID qdNanPoly1) was an adult female collected from Midlothian, Scotland, UK (latitude 55.93, longitude −3.05) on 2020-09-04. The specimen was collected by Olga Sivell and Duncan Sivell (both Natural History Museum), identified by Duncan Sivell and preserved in liquid nitrogen.

The specimen used for RNA sequencing (specimen ID NHMUK014447072, ToLID qdNanPoly4) was an adult female collected from Plymouth, England, UK (latitude 50.39, longitude −4.14) on 2020-09-20. The specimen was collected by Richard and Kenzou Twitchett (Natural History Museum), identified by Gregory Edgecombe (Natural History Museum) and then preserved in liquid nitrogen.

The initial identification was verified by an additional DNA barcoding process according to the framework developed by Twyford *et al*. (2024). A small sample was dissected from each specimen and stored in ethanol, while the remaining parts were shipped on dry ice to the Wellcome Sanger Institute (WSI) ( Pereira *et al.*, 2022). The tissue was lysed, the COI marker region was amplified by PCR, and amplicons were sequenced and compared to the BOLD database, confirming the species identification ( Crowley *et al.*, 2023). Following whole genome sequence generation, the relevant DNA barcode region was also used alongside the initial barcoding data for sample tracking at the WSI ( Twyford *et al.*, 2024). The standard operating procedures for Darwin Tree of Life barcoding have been deposited on protocols.io ( Beasley *et al.*, 2023).

### Nucleic acid extraction

The workflow for high molecular weight (HMW) DNA extraction at the Wellcome Sanger Institute (WSI) Tree of Life Core Laboratory includes a sequence of procedures: sample preparation and homogenisation, DNA extraction, fragmentation and purification. Detailed protocols are available on protocols.io ( Denton *et al.*, 2023b). The qdNanPoly8 sample was prepared for DNA extraction by weighing and dissecting it on dry ice ( Jay *et al.*, 2023). Tissue from the whole organism was homogenised using a PowerMasher II tissue disruptor ( Denton *et al.*, 2023a). HMW DNA was extracted using the Automated MagAttract v1 protocol ( Sheerin *et al.*, 2023). For ultra-low input (ULI) PacBio sequencing, DNA was fragmented using the Covaris g-TUBE method ( Oatley *et al.*, 2023). Sheared DNA was purified by solid-phase reversible immobilisation, using AMPure PB beads to eliminate shorter fragments and concentrate the DNA ( Strickland *et al.*, 2023). The concentration of the sheared and purified DNA was assessed using a Nanodrop spectrophotometer and a Qubit Fluorometer using the Qubit dsDNA High Sensitivity Assay kit. The fragment size distribution was evaluated by running the sample on the FemtoPulse system.

RNA was extracted from mid-body tissue of qdNanPoly4 in the Tree of Life Laboratory at the WSI using the RNA Extraction: Automated MagMax™ *mir*Vana protocol ( do Amaral *et al.*, 2023). The RNA concentration was assessed using a Nanodrop spectrophotometer and a Qubit Fluorometer using the Qubit RNA Broad-Range Assay kit. Analysis of the integrity of the RNA was done using the Agilent RNA 6000 Pico Kit and Eukaryotic Total RNA assay.

### Hi-C sample preparation and crosslinking

Hi-C data were generated from frozen tissue from the head and anterior body of the qdNanPoly1 sample, using the Arima-HiC v2 kit (Arima Genomics). As per manufacturer’s instructions, tissue was fixed, and the DNA crosslinked using a TC buffer with a final formaldehyde concentration of 2%. The tissue was then homogenised using the Power Masher-II (Diagnocine). The crosslinked DNA was digested using a restriction enzyme master mix, then biotinylated and ligated. A clean up was performed with SPRIselect beads prior to library preparation. DNA concentration was quantified using the Qubit Fluorometer v4.0 (Thermo Fisher Scientific) and Qubit HS Assay Kit and sample biotinylation percentage was estimated using the Arima-HiC v2 QC beads.

### Library preparation and sequencing

Library preparation and sequencing were performed at the WSI Scientific Operations core.

***PacBio HiFi (ULI)***

A ULI library was prepared using PacBio SMRTbell ^®^ Express Template Prep Kit 2.0 and PacBio SMRTbell ^®^ gDNA Sample Amplification Kit. To begin, samples were normalised to 20 ng of DNA. Initial removal of single-strand overhangs, DNA damage repair, and end repair/A-tailing were performed per manufacturer’s instructions. From the SMRTbell ^®^ gDNA Sample Amplification Kit, amplification adapters were then ligated. A 0.85X pre-PCR clean-up was performed with Promega ProNex beads and the sample was then divided into two for a dual PCR. PCR reactions A and B each followed the PCR programs as described in the manufacturer’s protocol. A 0.85X post-PCR clean-up was performed with ProNex beads for PCR reactions A and B and DNA concentration was quantified using the Qubit Fluorometer v4.0 (Thermo Fisher Scientific) and Qubit HS Assay Kit and fragment size analysis was carried out using the Agilent Femto Pulse Automated Pulsed Field CE Instrument (Agilent Technologies) and gDNA 55 kb BAC analysis kit. PCR reactions A and B were then pooled, ensuring the total mass was ≥500 ng in 47.4 μl. The pooled sample then repeated the process for DNA damage repair, end repair/A-tailing and additional hairpin adapter ligation. A 1X clean-up was performed with ProNex beads and DNA concentration was quantified using the Qubit and fragment size analysis was carried out using the Agilent Femto Pulse Automated Pulsed Field CE Instrument (Agilent Technologies). Size selection was performed using the PippinHT system (Sage Science) with target fragment size determined by analysis from the Femto Pulse, usually a value between 4000 and 9000 bp. Size-selected libraries were then cleaned-up using1.0X ProNex beads and normalised to 2 nM before proceeding to sequencing.

The sample was sequenced on a Revio instrument (Pacific Biosciences). The prepared library was normalised to 2 nM, and 15 μL was used for making complexes. Primers were annealed and polymerases bound to generate circularised complexes, following the manufacturer’s instructions. Complexes were purified using 1.2X SMRTbell beads, then diluted to the Revio loading concentration (200–300 pM) and spiked with a Revio sequencing internal control. The sample was sequenced on a Revio 25 M SMRT cell. The SMRT Link software (Pacific Biosciences), a web-based workflow manager, was used to configure and monitor the run and to carry out primary and secondary data analysis.

***Hi-C library preparation, amplification and sequencing***

Biotinylated DNA constructs were fragmented using the Covaris E220 sonicator (Covaris) and size-selected using SPRISelect beads to 400–600 bp. The DNA was enriched using the Arima-HiC v2 kit Enrichment beads. The NEBNext Ultra II DNA Library Prep Kit (New England Biolabs) was used for end repair, dA-tailing, and adapter ligation. This follows a modified NEBNext Ultra II DNA Library Prep protocol where library preparation occurs while DNA is bound to the Enrichment beads. Library PCR amplification was carried out using KAPA HiFi HotStart mix and a custom IDT UDI (Unique Dual Index) 96 barcode plate (Integrated DNA Technologies). Depending on the sample concentration and biotinylation percentage determined at the crosslinking stage, samples were run for 10–16 PCR cycles. Post-PCR, samples were cleaned up using SPRISelect beads. The libraries were quantified using the Accuclear Ultra High Sensitivity dsDNA Standards Assay kit (Biotium) and normalised to 10 ng/μL before sequencing. Hi-C sequencing was performed an Illumina NovaSeq 6000 instrument using paired-end sequencing with a read length of 150 bp.

***RNA***

Poly (A) RNA-Seq libraries were prepared using the NEBNext ^®^ Ultra™ II Directional RNA Library Prep Kit for Illumina (New England Biolabs), following the manufacturer’s instructions. Poly (A) mRNA in the total RNA solution was isolated using oligo (dT) beads, converted to cDNA, and uniquely indexed; 14 PCR cycles were performed. Libraries were size-selected to produce fragments between 100–300 bp. Libraries were quantified, normalised, pooled to a final concentration of 2.8 nM, and diluted to 150 pM for loading. Sequencing was carried out on the Illumina NovaSeq 6000 instrument.

### Genome assembly, curation and evaluation

***Assembly***

Prior to assembly of the PacBio HiFi reads, a database of *k*-mer counts ( *k* = 31) was generated from the filtered reads using FastK. GenomeScope2 ( Ranallo-Benavidez *et al.*, 2020) was used to analyse the *k*-mer frequency distributions, providing estimates of genome size, heterozygosity, and repeat content.

The HiFi reads were first assembled using Hifiasm ( Cheng *et al.*, 2021) with the --primary option. The Hi-C reads ( Rao *et al.*, 2014) were mapped to the primary contigs using bwa-mem2 ( Vasimuddin *et al.*, 2019), and the contigs were scaffolded using YaHS ( Zhou *et al.*, 2023) using the --break option for handling potential misassemblies. The scaffolded assemblies were evaluated using Gfastats ( Formenti *et al.*, 2022), BUSCO ( Manni *et al.*, 2021) and MERQURY.FK ( Rhie *et al.*, 2020).

The mitochondrial genome was assembled using MitoHiFi ( Uliano-Silva *et al.*, 2023), which runs MitoFinder ( Allio *et al.*, 2020) and uses these annotations to select the final mitochondrial contig and to ensure the general quality of the sequence.

***Assembly curation***

The assembly was decontaminated using the Assembly Screen for Cobionts and Contaminants (ASCC) pipeline. Flat files and maps used in curation were generated via the TreeVal pipeline ( Pointon *et al.*, 2023). Manual curation was conducted primarily in PretextView ( Harry, 2022) and HiGlass ( Kerpedjiev *et al.*, 2018), with additional insights provided by JBrowse2 ( Diesh *et al.*, 2023). Scaffolds were visually inspected and corrected as described by Howe *et al*. (2021). Any identified contamination, missed joins, and mis-joins were amended, and duplicate sequences were tagged and removed. The curation process is documented at https://gitlab.com/wtsi-grit/rapid-curation .

***Assembly quality assessment***

The Merqury.FK tool ( Rhie *et al.*, 2020), run in a Singularity container ( Kurtzer *et al.*, 2017), was used to evaluate *k*-mer completeness and assembly quality for the primary and alternate haplotypes using the *k*-mer databases ( *k* = 31) computed prior to genome assembly. The analysis outputs included assembly QV scores and completeness statistics.

A Hi-C contact map was produced for the final version of the assembly. The Hi-C reads were aligned using bwa-mem2 ( Vasimuddin *et al.*, 2019) and the alignment files were combined using SAMtools ( Danecek *et al.*, 2021). The Hi-C alignments were converted into a contact map using BEDTools ( Quinlan & Hall, 2010) and the Cooler tool suite (Abdennur & Mirny, 2020). The contact map was visualised in HiGlass ( Kerpedjiev *et al.*, 2018).

The genome was analysed using the BlobToolKit pipeline, a Nextflow ( Di Tommaso *et al.*, 2017) implementation of the earlier Snakemake BlobToolKit pipeline ( Challis *et al.*, 2020). The pipeline aligns PacBio reads using minimap2 ( Li, 2018) and SAMtools ( Danecek *et al.*, 2021) to generate coverage tracks. Simultaneously, it queries the GoaT database ( Challis *et al.*, 2023) to identify relevant BUSCO lineages and runs BUSCO ( Manni *et al.*, 2021). For the three domain-level BUSCO lineages, BUSCO genes are aligned to the UniProt Reference Proteomes database ( Bateman *et al.*, 2023) using DIAMOND blastp (Buchfink *et al.*, 2021). The genome is divided into chunks based on the density of BUSCO genes from the closest taxonomic lineage, and each chunk is aligned to the UniProt Reference Proteomes database with DIAMOND blastx. Sequences without hits are chunked using seqtk and aligned to the NT database with blastn ( Altschul *et al.*, 1990). The BlobToolKit suite consolidates all outputs into a blobdir for visualisation.

The BlobToolKit pipeline was developed using nf-core tooling ( Ewels *et al.*, 2020) and MultiQC ( Ewels *et al.*, 2016), with package management via Conda and Bioconda ( Grüning *et al.*, 2018), and containerisation through Docker ( Merkel, 2014) and Singularity ( Kurtzer *et al.*, 2017).

Table 4 contains a list of relevant software tool versions and sources. The Tree of Life pipelines can be accessed via this page: https://pipelines.tol.sanger.ac.uk/pipelines.

**Table 4.** Software tools: versions and sources.

Software tool	Version	Source
BLAST	2.14.0	ftp://ftp.ncbi.nlm.nih.gov/blast/executables/blast+/
BlobToolKit	4.3.9	https://github.com/blobtoolkit/blobtoolkit
BUSCO	5.5.0	https://gitlab.com/ezlab/busco
bwa-mem2	2.2.1	https://github.com/bwa-mem2/bwa-mem2
DIAMOND	2.1.8	https://github.com/bbuchfink/diamond
fasta_windows	0.2.4	https://github.com/tolkit/fasta_windows
FastK	666652151335353eef2fcd58880bcef5bc2928e1	https://github.com/thegenemyers/FASTK
Gfastats	1.3.6	https://github.com/vgl-hub/gfastats
GoaT CLI	0.2.5	https://github.com/genomehubs/goat-cli
Hifiasm	0.19.8-r603	https://github.com/chhylp123/hifiasm
HiGlass	44086069ee7d4d3f6f3f0012569789ec138f42b84aa44357826c0b6753eb28de	https://github.com/higlass/higlass
MerquryFK	d00d98157618f4e8d1a9190026b19b471055b22e	https://github.com/thegenemyers/MERQURY.FK
Minimap2	2.24-r1122	https://github.com/lh3/minimap2
MitoHiFi	3	https://github.com/marcelauliano/MitoHiFi
MultiQC	1.14, 1.17, and 1.18	https://github.com/MultiQC/MultiQC
Nextflow	23.10.0	https://github.com/nextflow-io/nextflow
PretextView	0.2.5	https://github.com/sanger-tol/PretextView
samtools	1.19.2	https://github.com/samtools/samtools
sanger-tol/ascc	-	https://github.com/sanger-tol/ascc
sanger-tol/blobtoolkit	0.6.0	https://github.com/sanger-tol/blobtoolkit
Seqtk	1.3	https://github.com/lh3/seqtk
Singularity	3.9.0	https://github.com/sylabs/singularity
TreeVal	1.2.0	https://github.com/sanger-tol/treeval
YaHS	1.2a.2	https://github.com/c-zhou/yahs

### Wellcome Sanger Institute – Legal and Governance

The materials that have contributed to this genome note have been supplied by a Darwin Tree of Life Partner. The submission of materials by a Darwin Tree of Life Partner is subject to the **‘Darwin Tree of Life Project Sampling Code of Practice’**, which can be found in full on the Darwin Tree of Life website here. By agreeing with and signing up to the Sampling Code of Practice, the Darwin Tree of Life Partner agrees they will meet the legal and ethical requirements and standards set out within this document in respect of all samples acquired for, and supplied to, the Darwin Tree of Life Project.

Further, the Wellcome Sanger Institute employs a process whereby due diligence is carried out proportionate to the nature of the materials themselves, and the circumstances under which they have been/are to be collected and provided for use. The purpose of this is to address and mitigate any potential legal and/or ethical implications of receipt and use of the materials as part of the research project, and to ensure that in doing so we align with best practice wherever possible. The overarching areas of consideration are:

• Ethical review of provenance and sourcing of the material

• Legality of collection, transfer and use (national and international)

Each transfer of samples is further undertaken according to a Research Collaboration Agreement or Material Transfer Agreement entered into by the Darwin Tree of Life Partner, Genome Research Limited (operating as the Wellcome Sanger Institute), and in some circumstances other Darwin Tree of Life collaborators.

## Author information

Members of the Natural History Museum Genome Acquisition Lab are listed here: https://doi.org/10.5281/zenodo.12159242.

Members of the Darwin Tree of Life Barcoding collective are listed here: https://doi.org/10.5281/zenodo.12158331.

Members of the Wellcome Sanger Institute Tree of Life Management, Samples and Laboratory team are listed here: https://doi.org/10.5281/zenodo.12162482.

Members of Wellcome Sanger Institute Scientific Operations: Sequencing Operations are listed here: https://doi.org/10.5281/zenodo.12165051.

Members of the Wellcome Sanger Institute Tree of Life Core Informatics team are listed here: https://doi.org/10.5281/zenodo.12160324.

Members of the Tree of Life Core Informatics collective are listed here: https://doi.org/10.5281/zenodo.12205391.

Members of the Darwin Tree of Life Consortium are listed here: https://doi.org/10.5281/zenodo.4783558.

## Data Availability

European Nucleotide Archive: Nanogona polydesmoides. Accession number PRJEB80243; https://identifiers.org/ena.embl/PRJEB80243. Thegenome sequence is released openly for reuse. The *Nanogona polydesmoides* genome sequencing initiative is part of the Darwin Tree of Life Project (PRJEB40665) and Sanger Institute Tree of Life Programme (PRJEB43745). All raw sequence data and the assembly have been deposited in INSDC databases. The genome will be annotated using available RNA-Seq data and presented through the Ensembl pipeline at the European Bioinformatics Institute. Raw data and assembly accession identifiers are reported in Table 1 and Table 2.
