# Supplementary material for: The genome sequence of the eyed flat-backed millipede, Nanogona polydesmoides (Leach, 1814)
Source: Wellcome Open Res. 2026 Oct 15;10:332. Originally published 2025 Jul 8. [Version 2] doi: 10.12688/wellcomeopenres.24341.2 (PMC12334921; doi:10.12688/wellcomeopenres.24341.2)
Supplement: Peer review history

# Reviewer report: Reviewer response for version 1

**DOI:** 10.21956/wellcomeopenres.26842.r127633

**Published:** 2025-08-08

**Authors:** Bruce A Snyder

**Affiliations:** 1 Georgia College & State University, Milledgeville, GA, USA

This is a well-written article describing the creation of a genome assembly for *Nanogona polydesmoides* via PacBio HiFi, Illumina Hi-C, and Illumina RNA-seq, and following well-established protocols and workflows.

 My only criticism is that Figure 1 is a poor representation of the species, showing only the ventral view of the posterior third of the body.

Are sufficient details of methods and materials provided to allow replication by others?

Yes

Is the rationale for creating the dataset(s) clearly described?

Yes

Are the datasets clearly presented in a useable and accessible format?

Yes

Are the protocols appropriate and is the work technically sound?

Yes

Reviewer Expertise:

Millipede biology, ecology, and taxonomy

I confirm that I have read this submission and believe that I have an appropriate level of expertise to confirm that it is of an acceptable scientific standard.

# Reviewer report: Reviewer response for version 1

**DOI:** 10.21956/wellcomeopenres.26842.r127630

**Published:** 2025-08-08

**Authors:** Wai Lok So

**Affiliations:** 1 The Chinese University of Hong Kong, Hong Kong, China

The authors sequenced the genome of *Nanogona polydesmoides, *which is the only British species of the genus. This genome enhances the millipede genomic database, with the addition of a Chordeumatida species. The sequencing method is sound and valid, performing in standard procedures. The procedures are also clearly written and described.

 Methods:

 - Please kindly provide more details on the specimen, including the sex, the stage and whether the gut of the sample has been removed before DNA isolation process.

 - Similar information should also be clearly written for samples used for RNA sequencing.

 Figure:

 Figure 1 - Any dorsal view of the specimen to include in the figure? Please do also add a scale bar on the figure.

Are sufficient details of methods and materials provided to allow replication by others?

Yes

Is the rationale for creating the dataset(s) clearly described?

Yes

Are the datasets clearly presented in a useable and accessible format?

Yes

Are the protocols appropriate and is the work technically sound?

Yes

Reviewer Expertise:

myriapod, millipedes, centipedes, genomics, evolution

I confirm that I have read this submission and believe that I have an appropriate level of expertise to confirm that it is of an acceptable scientific standard.
